# Dental Hygiene

**Published:** 1869-06

**Authors:** H. L. Ambler


### DENTAL REGISTER.

VOL. XXIII.] JUNE, 1869. [No. 6.

### Original Communications.

### DENTAL HYGIENE.

BY H. L. AMBLER, D. D. S., M. D.

Ovid, in addressing a beautiful lady, remarks : I can perceive your attention to the graces by the whiteness of your teeth. Lord Chesterfield says, that fine and clean teeth are among the first recommendations to be met with in the common intercourse of society. Thus we see, that both in ancient and modern times, the cleanliness of these organs was regarded as of the first importance; and in order to preserve their beauty to an old age, we must first consider their hygiene, which includes several subjects for our examination. First, some of the articles of food which are injurious: in general, animal substances are not as favorable to the preservation of the teeth as vegetable; for instance, there is more difficulty in removing the fibres of roast beef from between the teeth, or to remove the glutinous deposits of boiled meats, than there would be to get rid of vegetable accumulations. Also, the constant and prolonged use of smoked and salt meats, exert an unfavorable action on the gums, causing them to bleed easily, and the teeth to become loose and painful. Most people regard sugar as very injurious, but we should be inclined to doubt it, if we take the negroes for example, who, during a certain season of the year, eat almost nothing else, and they fatten on this diet; neither do they manifest any loss of the remarkable whiteness of their teeth. Although chemical analysis does not detect in sugar any acid quality, it is certain that most persons who eat largely of confectionery, experience sensations about the teeth, by no means favorable to the continuance of this indulgence. Workmen in sugar refineries generally have poor teeth, as do many of the girls employed in confectionery shops. This probably results from their partaking so often of the sweets, that some remains of them are left between the teeth, and undergoing a process of fermentation, have a very strong tendency to become acid, which certainly is injurious. If the chemical action of alcoholic drinks are not directly injurious to the teeth, they keep the mouth and gums in a state of irritation, which necessarily affects the teeth. River water is better for the teeth than well water, and in towns where no soft water can be procured, the inhabitants lose their teeth at the fortieth year. Young ladies, and also a great many older ones, often have an invincible propensity to devour acid substances, especially pickles, with the idea that it will give them a fine white complexion. We can not too frequently set before them the danger to their health, which such indulgence involves, and the destruction of their teeth, to them a priceless ornament, which must result if they should not suppress this depraved appetite.

A lady patient of mine, was advised by her physician, for some morbid affection, to eat lemons, which are mostly composed of citric acid; when she commenced, her teeth were good, and of a bluish white color, but in one week, owing to the action of such a powerful acid on the tooth structure, they were completely whitened, and she was obliged to discontinue this treatment, as she could not eat anything with comfort; for, said she, my teeth are all on edge. Gargles of nitric acid are sometimes prescribed for affections of the throat, but in the use of this or any other acid in connection with the mouth, we should be careful to thoroughly cleanse the teeth immediately afterward, and an alkaline preparation is the proper thing to use; as it is these acids that are the immediate cause of Dental caries. Of all the forms of vegetable matter in this climate, the farinaceous grains are the most important. But in making them into bread, the coarse part, which is the tooth building substance, is separated from the fine white portion and thrown aside. In the inner skin of wheat there is an oily quality of a sweet nature, and bread made of both the coarse and fine portions is sweeter, easier digested, and better in every way for the support of the teeth, as well as the general health. Barlow says:

"To mix the food by vicious rules of art,
To kill the stomach and to sink the heart,
To make mankind in social virtue sour,
Cram o'er each dish, and be what they devour;
For this the kitchen nurse first framed her book,
Commanding sweat to stream from every cook,
Children no more their antic gambols tried,
And friends to physic wondered why they died."

We will now notice some of the influences of clothing and atmospheric changes. Next to the lungs the teeth are the most exposed to suffering from the daily imprudences committed in these respects. They may be affected directly or indirectly. Directly by the sharp stimulation which the cold imparts to the blood vessels and nerves enclosed by the membrane of the dental canal and pulp chamber. Indirectly, by the sudden suppression of transpiration from some part of the body, which is thrown upon the mucous membrane of the mouth, and thence upon the teeth, causing inflammation. Ladies especially, on account of their organizations, are most sensible to slight changes of temperature. The best means of protection is to acquire early the habit of not clothing the person too heavily, and to take exercise in the open air. This will favor the harmonious development of all parts of the body, and give to each the power to resist morbid influences. As soon as there is a change of temperature, we should take care to clothe ourselves suitably, particularly when passing out into the air from crowded rooms, where the exhilaration of pleasure and the high temperature has excited us to the utmost. Among the things hurtful to the teeth, we notice the bad habit of using them for purposes for which they were never intended. Persons, who with their teeth crack nuts, draw corks and nails, lift heavy weights, and bite thread, a thing especially to caution the ladies against, only expose to premature decay, organs indispensable to nutrition and beauty. Inveterate smoking is also to be deprecated, for it corrodes the teeth, and the sudden change many times in inhaling cold air, causes an inflammatory action of the mucous membrane of the mouth. The continued use of pipes and cigar holders, being made of hard substances, wear away the teeth. Look at an old man who smokes a clay pipe for an example, and you will find the lateral incisor and cuspid worn to such a shape, that they exactly fit the stem of his pipe. There is a habit which the ladies have of putting pins and needles in their mouth, and often carrying them there for a long time. This is no little matter, for the contact of these hard bodies, pressed with more or less force will wear away the enamel, and sometimes induce caries of the whole tooth.

One of the simplest means of preserving the teeth, consists in cleanliness of the mouth. The first thing after rising in the morning, or from a meal, should be to cleanse the mouth thoroughly with tepid water. It is the custom in some parts of England and France, to rinse the mouth with warm aromatic water after eating. It is well to remember that this precaution not only tends to keep the teeth clean, but to clear the voice of those about to sing or converse. By cleansing the teeth three times a day regularly, the formation of tartar is not only prevented, but such particles of food and other extraneous matter as lodge about and adhere to them, causing irritation and inflammation are by this means removed. The fermentation of vegetable substances in the mouth, produces indirectly sulphuric acid. Animal and nitrogenous substances producing nitric acid. These vitiate the fluids of the mouth and help the teeth on to certain decay. Attention to cleanliness of the teeth in early life, can not be too urgently insisted upon, for it is evident that most of their diseases arise from foreign matter being suffered to remain upon and between them, and no time therefore should be lost, in removing what has accumulated as soon as it is discovered.

From what we know of the nature and use of the teeth, it is evident that whatever is capable of destroying them, must be injurious in the highest degree. All acids, gritty powders, and tinctures, that for a time give whiteness to the teeth, are certain ultimately to destroy their texture, and render them more liable to decay. The way to preserve the color of the teeth, is to remove whatever may collect upon them, and thus allow them to possess their natural whiteness and polish. The best method to effect this is with a brush and tepid water, then pass a thread of waxed floss silk between them, to dislodge whatever may have collected on their approximal sides. If these means do not subserve to prevent the accumulation of tartar, we would recommend as the most simple dentifrice, a nice article of precipitated chalk, which possesses alkaline properties sufficient to help neutralize the fluids of an acid character which come in contact with the teeth, and to promote their well-being, as well as the parts surrounding them. The habit which many have of scouring their teeth with soot or charcoal, is a detestable practice. The small black grains remain between the necks of the teeth and the gums, and their constant use in many cases will cause absorption of the gums around the necks of the teeth; and beside all this, they scratch the enamel. This will seem very plain, when we remember that charcoal is used for polishing steel.

If we take a survey of society in general, we shall be forced to conclude, that perfect cleanliness of the mouth and teeth, is considered as an object of minor importance, and the attention of parents can not be too closely directed to this fact. It is said that habit is second nature, therefore practice should be so modified to the nature and the ability of the individual being, as to produce upon him with uniformity, a profitable and beneficial result. The knowledge of using the means to keep the mouth and teeth cleansed, should be possessed by every mother, as society imposes upon her the task of directing the early habits and impressions of her children. If parents are brought to reflect upon this subject in relation to the importance it bears to the future health and happiness of their offspring, and avail themselves of the best information on this topic, so interwoven with the best interests of society; how much good would be dispensed and how extended the beauty and happiness of mankind.

In regard to the care of children's teeth, we would say: As soon as the teeth make their appearance, it should be the duty of the mother or nurse, to clean them morning and evening with a small brush and tepid water, and as they increase in number, floss silk well waxed should be passed between them, moving it up and down a little under the gums, for the purpose of removing all accumulations, remembering that food, fruit, etc , left in the mouth and between the teeth during sleep, are the principal causes of their decay. Early and careful attention to the teeth, cleanliness of mouth, temperance in living, and abstinence from acids, are some of the best maxims for the preservation and beauty of the teeth. When children are thus familiarized to the healthy and necessary custom of brushing the teeth, it becomes a fixed habit, and they will find it ever afterwards absolutely essential to their comfort. As soon as the child is old enough, give it a tooth brush, and give instructions for its use, and see that it is done often and thoroughly. The brushes to be used should be adapted to each case, neither too soft nor too hard, and so formed as to clean the teeth without injuriously irritating the surrounding tissues. Brushes for children should not be quite as stiff as for older persons, the gums not having been subjected to as much friction, are not so dense. Procure brushes of a medium width, and narrow at their extremity, so as easily to penetrate to the last molars without wounding the cheeks; they should have three rows of bristles, with the handle slightly bent, so as to allow of an easy and graceful motion. In conclusion we would say:

> "Let every fair one shun Urilla's fate,
> And wake to action ere it be too late;
> Let each successive day unfailing bring
> The brush, the dentifrice, and from the spring
> The cleansing flood, the labor will be small,
> And blooming health will soon reward it all;
> Or if her past neglect preclude relief,
> By gentle means like these assuage her grief;
> The dental art can remedy the ill,
> Restore her hopes and make her lovely still."

